# Kelp and dolphin gulls cause perineal wounds in South American fur seal pups (*Arctocephalus australis*) at Guafo Island, Chilean Patagonia

**DOI:** 10.1098/rsos.170638

**Published:** 2017-07-26

**Authors:** Mauricio Seguel, Francisco Muñoz, Felipe Montalva, Diego Perez-Venegas, Héctor Pavés, Nicole Gottdenker

**Affiliations:** 1 Department of Pathology, University of Georgia, College of Veterinary Medicine, Athens, GA, USA; 2 Instituto de Patología Animal, Facultad de Ciencias Veterinarias, Universidad Austral de Chile, Valdivia, Chile; 3 Facultad de Ciencias, Departamento de Biología Marina, Pontificia Universidad Católica de Chile, Santiago, Chile; 4 Conservation Medicine Program and Departamento de Ecología y Biodiversidad, Facultad de Ecología y Recursos Naturales, Universidad Andres Bello, 252 Republica St., Santiago, Chile; 5 Departamento de Ciencias Básicas, Universidad Santo Tomas, Los Carrera 753, Osorno, Chile

**Keywords:** *Arctocephalus australis*, dolphin gull, fur seal, hookworm, kelp gull, wound

## Abstract

During five reproductive seasons, we documented the presence, extent and origin of perineal wounds in South American fur seal pups (*Arctocephalus australis*) on Guafo Island, Northern Chilean Patagonia. The seasonal prevalence of perineal wounds ranged from 5 to 9%, and new cases were more common at the end of the breeding season (February), when pups were on average two months old and were actively expelling hookworms (*Uncinaria* sp). Histologically, wounds corresponded to marked ulcerative lymphoplasmacytic and histiocytic dermatitis with granulation tissue and mixed bacterial colonies. In 2015 and 2017, kelp gulls (*Larus dominicanus*) and dolphin gulls (*Leucophaeus scoresbii*) were observed picking and wounding the perineal area of marked pups. This behaviour occurred more frequently after the pups' defecation, when sea gulls engaged in consumption of pups' faeces. The affected pups usually had moderate to marked hookworm infections along with bloody diarrhoea and anaemia. Pups with severe wounds (23% of affected animals) had swollen perineal areas and signs of secondary systemic bacterial infection. We propose that seagulls on Guafo Island have learned to consume remains of blood and parasites in the faeces of pups affected by hookworm infection, causing perineal wounds during this process. We conclude that this perineal wounding is an unintentional, occasional negative effect of an otherwise commensal gull–fur seal relationship.

## 1. Introduction

Seagulls are important species in marine ecosystems, acting mostly as scavengers and/or predators [1,2]. Most seagull species show high plasticity in foraging behaviour, however, and can adapt their diet drastically depending on resource availability [3,4]. This foraging plasticity has led to the development of singular interactions between seagulls and other marine organisms, which go from the use of food resources captured by other animals (kleptoparasitism) to parasitism on whales [5,6]. In most pinniped reproductive colonies, seagulls are a common inhabitant along with other scavengers [7,8]. Their presence is mostly due to the large amount of food resources available, which includes placentas, carcasses of large territorial males that die during fights, and numerous dead pups due to the high neonatal mortality in many pinniped species [8,9]. Whereas these scavenging behaviours can be beneficial for the overall pinniped population, for example, by reducing the amount of debris and potential infectious material in the rookery, detrimental interactions for marine mammals have also been recorded. In Baja California, western gulls (*Larus occidentalis*) are thought to opportunistically predate on California sea lion pups (*Zalophus californianus*) when they consume placentas attached to the pups [10]. In Namibia, kelp gulls (*Larus dominicanus*) prey on the eyes of Cape fur seal pups (*Arctocephalus pusillus*) [2]. One of the most bizarre and well-recorded interactions between gulls and marine mammals is the parasitism of kelp gulls on adult and neonate Southern right whales (*Eubalaena australis*) at Peninsula Valdes, Argentina [11,12]. In this location, kelp gulls have learned over the course of 30 years, to open wounds in the skin and blubber of the whale's back to feed on those tissues when the whales reach the sea surface to breathe [11]. The effect of this wounding can be quite dramatic, and is thought to be one of the factors related to the high mortality of Southern right whale calves at Peninsula Valdes [11,12].

Guafo Island, located in the oceanic portion of the Northern Chilean Patagonia, harbours exuberant marine biodiversity, including the largest breeding colony of South American fur seals (*Arctocephalus australis*) in the Pacific Ocean and the largest colony of sooty shearwaters in the world [9,13]. Like most South American coasts, kelp gulls are common in Guafo Island, with many reproductive colonies throughout the north side [13,14]. Furthermore, dolphin gulls (*Leucophaeus scoresbii*) have important nesting sites on the northwest point of the island and are particularly numerous at the fur seal rookery [15]. As part of South American fur seal health monitoring programmes, pups have been captured, sexed, weighed and measured every reproductive season for the last 10 years. Since 2008, perineal wounds have been observed in the pups; however, the cause of these lesions has remained unknown.

In this paper, we summarize 5 years of observational and experimental studies confirming the role of kelp and dolphin gulls in the development of perineal wounds in South American fur seal pups. We also document the effect of perineal wounds on pup health status and propose an explanation for the seagull behaviour that causes perineal wounding.

## 2. Material and methods

This study was conducted in the reproductive colony of South American fur seals on Guafo Island (43.593029° S, 74.713481° W), Northern Chilean Patagonia, during the 2012 through 2017 Austral summers (10 December to 10 March). South American fur seal (SAFS) pups (*N* = 668) were captured by hand as part of routine health assessments. During capture procedures, the weight, total length and sex of each pup were recorded. Additionally, all pups underwent a quick physical examination by an experienced medical veterinarian to determine the overall health status and to record significant clinical findings such as: perineal wounds, purulent conjunctivitis, bloody diarrhoea, abscesses, coughing, fresh umbilical cord and the presence of mucus or dead parasites in the anus. Pups were considered to present bloody diarrhoea when they defecated liquid faeces with semi-digested blood during the capture and physical exam or when they presented a significant amount of semi-digested blood mixed with faeces in the faecal swabs collected for parasitology analyses. Perineal wounds were categorized as mild if there were two wounds or fewer of less than 2 cm in diameter each, and severe if there were more than two wounds (of any diameter) or at least one wound of more than 2 cm in diameter. All captured pups had blood extracted from the caudal gluteal vein, and complete blood cell counts and coproparasitologic exams were performed in the field laboratory as previously described [16]. In some pups (*n* = 8 of pups affected by perineal wounds), all blood cell count parameters could not be measured due to partial blood coagulation in the EDTA tube. All captured pups were marked with correlative numbers in the fur using commercial hair decolorant. During 2014, 2015 and 2017, most captured pups (50–70% of them) were recaptured every 10–20 days to record progress of perineal wounds and other health-related conditions (e.g. hookworm infection). To measure the effect of hookworm infection on pup survival, in 2017 a subset of pups (*n* = 30) was treated with the antiparasitic drug ivermectin (400 µg kg^−1^) early in the reproductive season, when pups were on average one week old (15–21 December) [17].

In 10 animals with perineal ulcers, sterile culture swabs were collected using a commercial swab transport media kit (BBL™ CultureSwab™, BD, New Jersey, USA). Swabs were stored at 4°C or −18°C for 4 or 30 days, respectively, until transported to the mainland laboratory where frozen swabs were thawed at room temperature for 12 h. Swabs were plated on blood and McConkey agars and incubated at 37°C for 48 h. Isolated bacterial colonies were identified to the genus or species level by Gram stain and biochemical reactions using a BBL Crystal ID system for enteric/non-fermenting and Gram-positive bacteria.

In five of the animals that underwent bacteriological culture of perineal wounds, biopsy samples were collected using a 4 mm commercial biopsy punch, after local administration of lidocaine 2% in the wounded area. Biopsy samples were stored in 10% buffered formalin and routinely processed for histopathology. Tissue slides were stained with haematoxylin and eosin (H&E), Gram, Gomori Methenamine Silver, Wharthin-Starry and Acid-Fast stains, and Periodic acid-Schiff reaction, and examined by two board-certified pathologists (MS, NG).

In 2015 and 2017, daily observations (3 h per day) of the Guafo Island fur seal rookery from elevated points allowed us to closely record the interaction of kelp and dolphin gulls with previously marked SAFS pups. The number of interactions was recorded and the main features of pup and sea gull behaviour were documented through written descriptions and photographs. To detect variations in sea gull populations at the fur seal rookery, we used photographs of the fur seal rookery, taken from elevated points and routinely used to estimate fur seal numbers (Perez-Venegas D, Paves H, Seguel M. 2015, unpublished data), to estimate the total number of sea gulls on different reproductive seasons (total number of kelp and dolphin gulls observed on 15 January).

Perineal wound prevalence was calculated as the total number of pups that presented perineal wounds in a reproductive season divided by the total number of pups sampled the same season. Differences in perineal wounds between seasons were tested by a generalized linear model (GLM) using individual years as a categorical explanatory variable of the presence of perineal wounds. The weekly incidence of perineal wound was calculated by combining all perineal wounds events and recording the corresponding week of the reproductive season when they were diagnosed. Data on pups without perineal wounds were selected by matching the date of capture of the pups that had perineal wounds (e.g. capture in January 2013, six animals with wounds and six without wounds) in order to determine the relationship between pup's biometric (body mass index) and health-related variables (haemoglobin concentration, total white blood cell (WBC) counts, the presence of bloody diarrhoea, etc.) and the presence of perineal wounds The group without wounds was used as control for comparative analyses through GLM. To determine the variables that better explained the presence of perineal wounds in a pup, all variables from pups with and without wounds were condensed in a single table (96 pups) and logistic generalized linear models were fitted (GLM, family = ‘binomial’) using the presence of perineal wounds as a response variable and haemoglobin concentration (Hg), number of hookworm eggs in faeces, body mass index, sex, WBC counts, number of band neutrophils, total protein (TP), number of lymphocytes, number of macrophages, the presence of conjunctivitis, anal mucus, bloody diarrhoea and coughing, and their interactions as predictors in the full model. Different models were fitted and several candidate models selected based on Akaike information criteria with correction for small sample size (AIC_c_, ‘MuMIn’ package, ‘R 3.2.0’ statistical software). Additionally, the models with a delta AIC_c_ < 7 when compared to the model with the lowest AIC_c_ were averaged and predictability compared with individual models through mean absolute error (MAE, table 2). Finally, inference was made based on information provided by the top and averaged models. The effect of antiparasitic treatment on the incidence of perineal wounds was assessed by comparing outcomes in treated and untreated groups using a *χ*^2^-test in a contingency table. The power of this experiment was calculated with the ‘pwr’ package of ‘R.3.2.0’ statistical software (R core development team) and using 0.1 as effect size, based on the known prevalence of perineal wounds in the studied population (approx. 10%). All statistical tests were performed using ‘R 3.2.0’ statistical software.

## 3. Results

**Figure 1.**
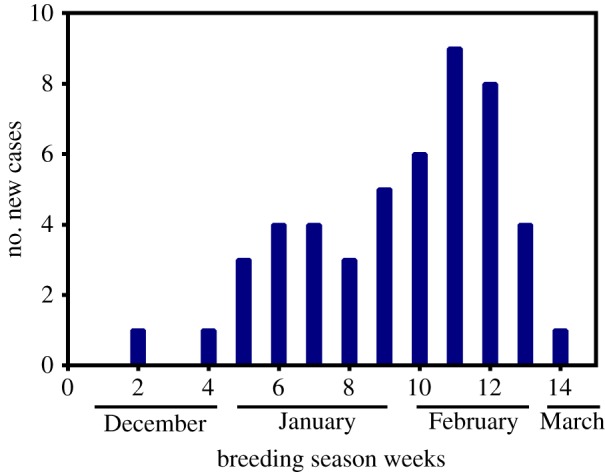
Incidence of perineal wounds in South American fur seal pups at Guafo Island. The majority of the new cases were registered during the third and fourth weeks of February when pups are on average two months old.

**Figure 2.**
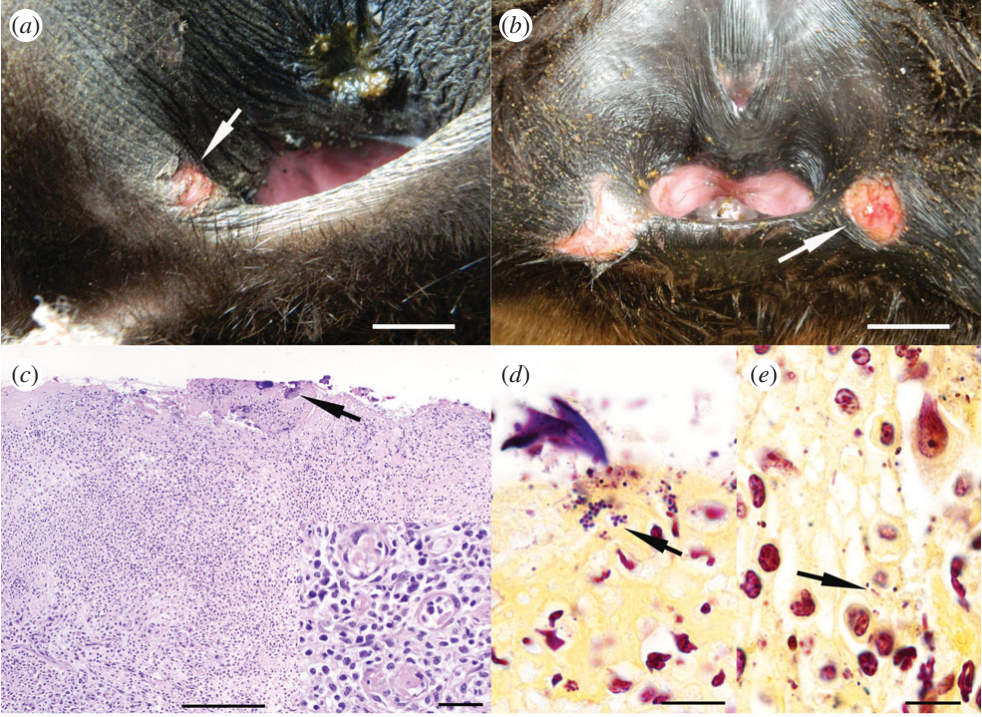
Macroscopic and histological features of perineal wounds in South American fur seal pups. (*a*) Perineal area of a female pup with a small laceration (mild perineal wound) at the margin of the vulvar vestibule (arrow). Scale bar, 0.5 cm. (*b*) Female pup with two large wounds (severe wounds) at the latero-ventral margin of the perineal area. Scale bar, 2.0 cm. (*c*) Perineal wound (H&E) composed by granulation tissue covered by fibrin, foreign material and bacteria colonies (arrow). Scale bar, 100 µm. Inset. Detail of granulation tissue composed by numerous new blood vessels, fibroblasts, macrophages, lymphocytes and plasma cells. Scale bar, 15 µm. (*d*) Gram stain of perineal wound showing colonies of Gram-positive cocci (arrow). Scale bar, 10 µm. (*e*) Gram stain of perineal wound showing intrahistiocytic Gram-negative bacilli and Gram-positive cocci (arrow). Scale bar, 10 µm.

The prevalence of perineal ulcers in SAFS pups ranged from 5 to 9% among reproductive seasons; however, prevalence differences were not significant (GLM, d.f. = 664, *p* > 0.24; table 1). Perineal wounds were more common later in the season, when pups were on average two months old, peaking on the second week of February (figure 1). The severity of the perineal lesions was homogeneous throughout seasons, with 70–80% of the cases consisting of one or two oval, and well demarcated ulcers of 1–2 cm in diameter (mild wounds; figure 2*a*). In the remaining 20–30% of cases, the lesions consisted of three to five ulcers in the perineal area, or a single ulcer of more than 2 cm in diameter (severe lesions; figure 2*b*). Pups with severe wounds (11 out of 47 pups affected by perineal wounds) had very inflamed perineal areas and were lethargic. Histologically, there was moderate to marked ulcerative lymphoplasmacytic and histiocytic dermatitis with marked proliferation of fibroblasts and small calibre new blood vessels (granulation tissue; figure 2*c*). We found small colonies of Gram-positive cocci and Gram-negative bacilli (figure 2*d*) on the edge of ulcerated areas, admixed with cellular debris and foreign material. Bacteriology of perineal ulcers yielded mixed growth of *Staphylococcus* sp., *Enterococcus faecalis*, *Escehrichia coli, Proteus* sp. and *Klebsiella* sp.

**Table 1.** Prevalence of perineal wounds in South American fur seal pups (*Arctocephalus australis*) at Guafo Island, Northern Chilean Patagonia. Differences were not significant at *α* = 0.05 (generalized linear model).

breeding season	no. animals with perineal wounds	no. animals sampled	prevalence (%)
2012	6	120	6
2013	7	130	5
2014	10	112	9
2015	6	94	6
2016	8	101	8
2017	10	111	9
Total	47	668	7

**Figure 3.**
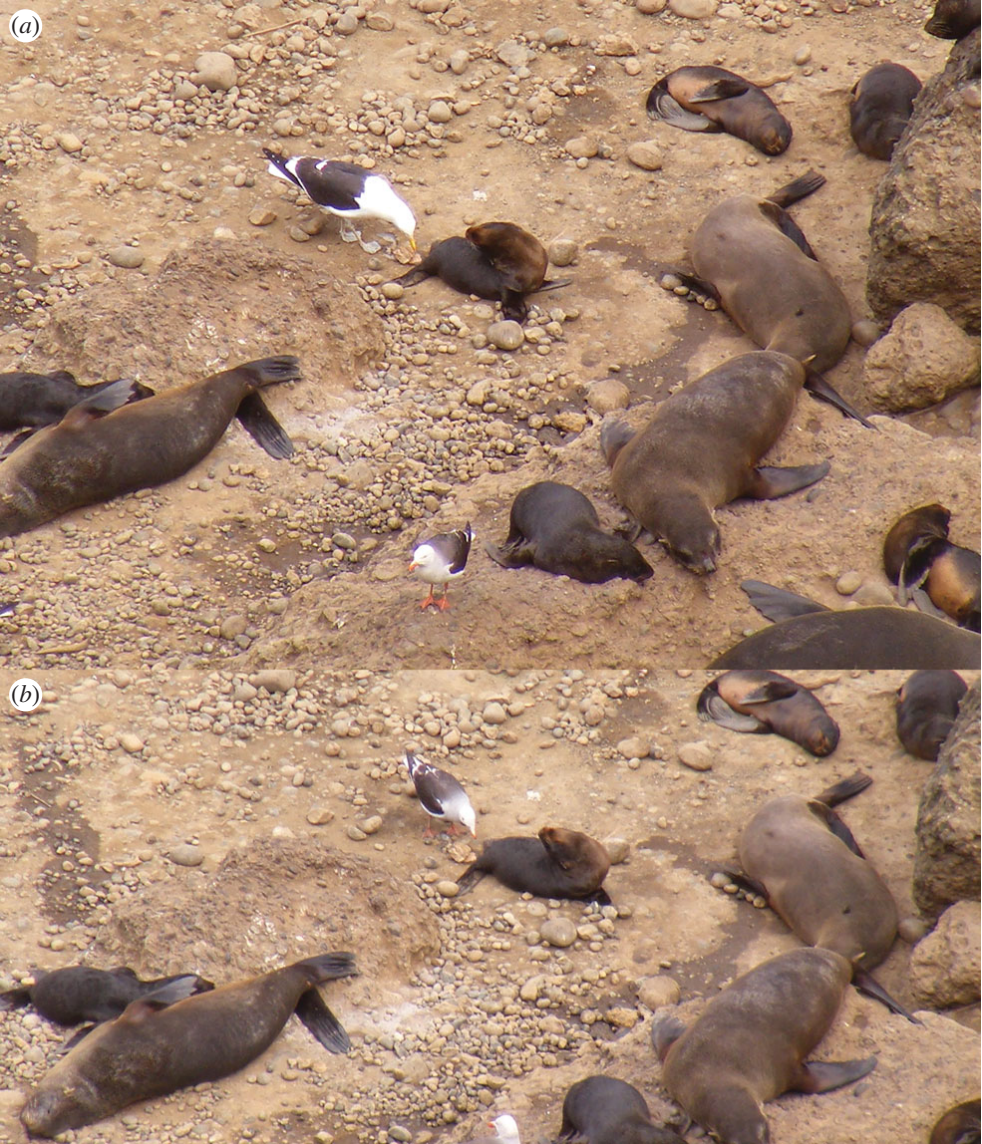
Seagulls picking the perineal area of South American fur seals at Guafo Island. (*a*) A kelp gull (*Larus dominicanus*) engages in the picking of a fur seal pup perineal area after this pup has defecated. A dolphin gull (*Leucophaeus scoresbii*) observes from a few metres away. (*b*) A dolphin gull eats rests of faeces and picks the perineum of the same fur seal pup showed in (*a*), after the kelp gull has left the area.

In 2015 and 2017, kelp and dolphin gulls were observed approaching fur seal pups and picking the perineal area on 20 occasions in a total of 160 observation events (208 h of observation). This behaviour usually occurred right after pup defecation, when kelp and dolphin gulls engaged in consumption of pup faeces and picked the perineal areas spotted with faecal material. Kelp gulls usually interacted first with fur seal pups. After kelp gulls had consumed some faeces or picked the perineal area three to five times, they retired, leaving pups vulnerable to interactions with dolphin gulls that would actively consume the remaining faeces and aggressively pick the perineal area (figure 3*a,b*). The response of pups was usually limited to moving away from the area or trying to bite seagulls, but they never succeeded in their attempts in any recorded interaction. On rare occasions (*n* = 6), dolphin gulls approached sleeping pups and picked the perineal region in a similar manner to how they approach dead pups and scavenged internal organs. On three occasions, marked pups that interacted with sea gulls were captured 1 or 2 days later, and we discovered they presented fresh perineal wounds that were absent on previous captures performed 5–7 days earlier. The number of kelp gulls at the Guafo Island fur seal rookery ranged from 33 to 40 animals between seasons and the number of dolphin gulls ranged from 71 to 84 individuals during different seasons, according to photographic censuses taken 15 January of every year (table 2).

**Table 2.** Number of kelp (*Larus dominicanus*) and dolphin (*Leucophaeus scoresbii*) gulls at the rookery of South American fur seals (*Arctocephalus australis*) in Guafo Island, Northern Chilean Patagonia.

breeding season	no. *Larus dominicanus*	no. *Leucophaeus scoresbii*
2012	40	78
2013	51	82
2014	39	75
2015	35	71
2016	33	78
2017	38	74

**Figure 4.**
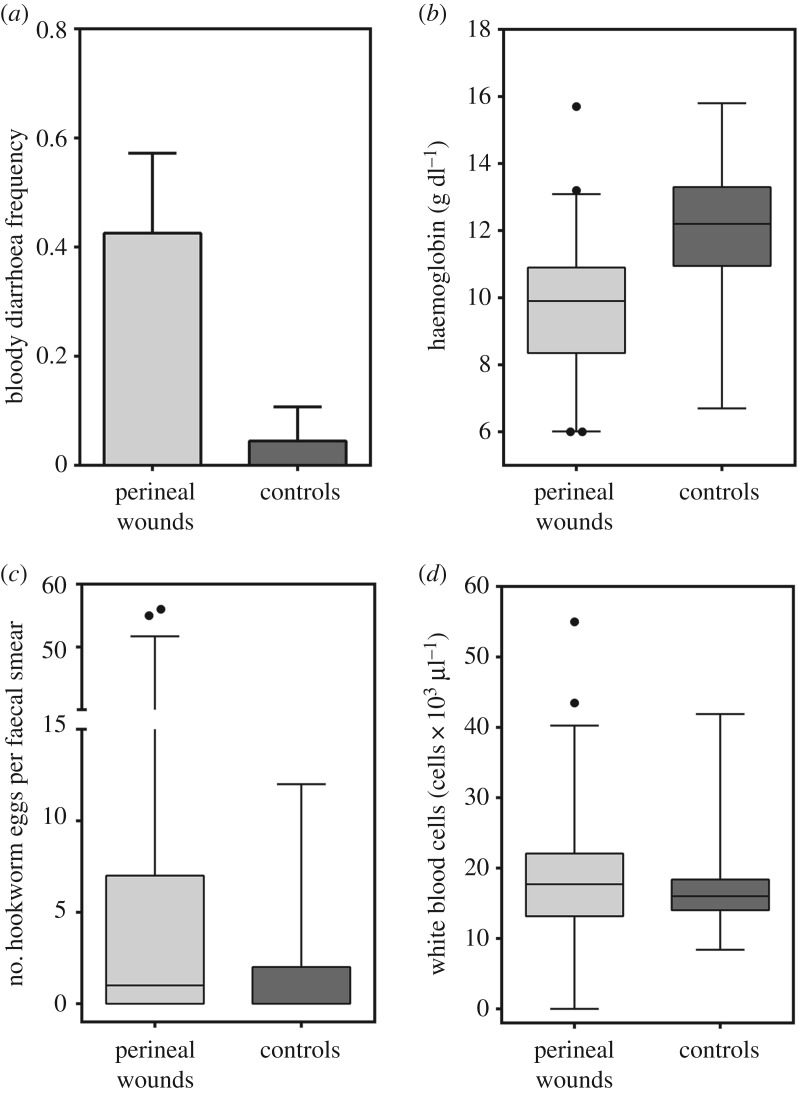
Differences in health parameters between pups with perineal ulcers and matched controls (pups without perineal ulcers). (*a*) Almost half of the pups with perineal wounds had bloody diarrhoea compared to only 4% of pups without perineal wounds (GLM, *Z* = 3.54, d.f. = 90, *p* < 0.001). (*b*) Pups with perineal wounds had lower haemoglobin concentration compared with controls (GLM, *Z* = −3.89, d.f. = 83, *p* < 0.001). (*c*) Pups with perineal wounds had higher numbers of hookworm eggs in their faeces compared with controls (GLM, *Z* = 2.01, d.f. = 84, *p* = 0.043). (*d*) Pups with perineal wounds had higher numbers of leucocytes in the peripheral blood when compared with controls (GLM, *Z* = 2.43, d.f. = 81, *p* = 0.015).

The most common co-morbidity at clinical examination of pups with perineal wounds was bloody diarrhoea, with 40% of pups with perineal wounds affected by this condition, compared with 4.4% of control pups (without perineal wounds; GLM, *Z* = 3.54, d.f. = 90, *p* < 0.001; figure 4*a*). Haemoglobin concentrations of pups with perineal wounds compared with control pups were significantly lower (GLM, *Z* = −3.89, d.f. = 83, *p* < 0.001; figure 4*b*). The inverse pattern was observed for hookworm (*Uncinaria* sp.) burden and number of WBCs, with animals affected by perineal wounds having a higher numbers of *Uncinaria* sp. eggs in the faeces (GLM, *Z* = 2.01, d.f. = 84, *p* = 0.043) and a higher number of leucocytes in the peripheral blood when compared to pups not affected by wounds (GLM, *Z* = 2.43, d.f. = 81, *p* = 0.015; figure 4*c,d*). This increase in leucocytes was attributed mostly to a rise in the number of circulating young (band) neutrophils (left shift; GLM, *Z* = 2.72, d.f. = 59, *p* < 0.006), while the numbers of mature neutrophils, lymphocytes, monocytes and eosinophils were comparable between pups with and without perineal wounds (GLMs, all *p* > 0.05). Differences in leucocyte profiles (leukocytosis with left shift), were even larger when only pups with severe wounds were compared with controls (severe perineal wounds range = 1.3–2.8 × 10^3^ bands µl^−1^, median = 2.1 × 10^3^ bands µl^−1^; controls range = 0.39–1.09 × 10^3^ bands µl^−1^, median = 0.92 × 10^3^ bands µl^−1^). According to the best set of logistic models, pups with a lower haemoglobin concentration and a higher number of band (immature) neutrophils were more likely to have perineal wounds (several GLM-binomial, *p* < 0.001; models and coefficients in tables 3 and 4). The effect of haemoglobin concentration was consistently the most significant, however, being present in all the models with low AIC_c_ scores (delta AIC_c_ < 7; table 3). Additional factors that were associated with an increased likelihood of perineal wounds included a higher body mass index and the presence of conjunctivitis; however, their effect was not significant at alpha 0.05 (table 4). Predictors of less importance, based on the number of models containing the factors, but with significant coefficients, included lower numbers of eosinophils and neutrophils, and the presence of blood in the faeces (table 4).

**Table 3.** Log likelihood (logLik), Akaike information Criteria corrected for small samples size (AIC_c_), AICc weight (AIC_CW_) and MAE for binomial models with the presence of perineal wounds as response. Hg, haemoglobin concentration; BMI, body mass index; Bands, number of bands; Conj., presence of conjunctivitis; WBC, total number of white blood cells; Mac, number of macrophages; Lymph, number of lymphocytes; Mucus, the presence of mucus; Eos, number of eosinophils; Blood.faeces, presence of blood in the faeces; Neut, number of neutrophils; TP, Total protein serum concentration.

model	predictors	d.f.	logLik	AIC_C_	delta AIC_C_	AIC_CW_	MAE
1	Hg + BMI + Bands + Conj. + Bands:WBC + Mac	7	−12.8993	42.5987	0	0.358088	0.148091
2	Hg + BMI + Bands + Conj. + Bands:WBC + Mucus:Lymph	7	−13.3765	43.55292	0.954225	0.22222	0.1678
3	Hg + BMI + Bands + Conj. + Mucus + Eos + Blood.faeces + Neut	9	−11.127	44.99078	2.39208	0.108282	0.1498
4	Hg + BMI + Bands + Mucus + Eos + Blood.faeces + Neut + Conj:WBC	9	−11.1542	45.0453	2.4466	0.10537	0.150264
5	Hg + BMI + Bands + Conj. + Bands:WBC + Mucus + WBC	8	−13.2844	46.26114	3.662445	0.057372	0.166484
6	Hg + BMI + Conj. + Bands:WBC + Mucus + Eos + Blood.faeces + Neut	9	−12.1105	46.95786	4.359166	0.040496	0.166209
7	Hg + BMI + Bands + Conj. + Bands:WBC	6	−16.8845	47.81783	5.219133	0.026343	0.212007
8	Hg + BMI + Bands + Conj. + Bands:WBC + Mucus + WBC:Conj + TP	9	−13.2176	49.17198	6.573282	0.013385	0.166126
9	Hg + BMI + Bands + Conj. + Bands:WBC + Blood.faeces:WBC	7	−16.2022	49.20437	6.605673	0.01317	0.197951
10	Hg + BMI + Bands + Conj. + Bands:WBC + Eos	7	−16.6858	50.17165	7.572947	0.00812	0.207919
average	all predictors					0.952845	0.154154

**Table 4.** Coefficients of 10 different binomial generalized linear models with presence or absence of perineal wounds in South American fur seal pups as response variable (*n* = 96) (models shown in table 3). ‘Average’ corresponds to the averaged coefficients for the 10 models.

	models										
predictors	1	2	3	4	5	6	7	8	9	10	average
intercept	8.213	5.326	12.768	12.685	6.848	14.378*	5.683	5.843	4.625	5.802	8.52
haemoglobin (g dl^−1^)	−1.284^**^	−1.130^**^	−1.313^**^	−1.306^**^	−1.165^**^	−1.324^**^	−0.959^**^	−1.142^**^	−0.878^**^	−0.943^**^	−1.23^**^
body mass index	39.876	46.070	56.766	56.664	45.569	52.812	31.449	44.078	32.350	33.120	45.76
bands (cells µl^−1^)	0.008^**^	0.008^**^	0.002	0.002	0.007^**^		0.007^**^	0.007*	0.007^**^	0.006^**^	0.006*
conjunctivitis	22.341	−4.285	−4.332		−4.396	−3.855	−4.012	−4.432	−4.136	−3.583	7.09
bands :WBC	−2.2 × 10^−7**^	−2.0 × 10^−7**^			−1.70 × 10^−7^	7.02 × 10^−8^	−1.8 × 10^−7**^	−1.60 × 10^−7^	−2.30 × 10^−7*^	−1.5 × 10^−7*^	−1.9 × 10^−7^
mucus			25.802	25.775	20.808	25.745		20.952			24.7
eosinophils (cells µl^−1^)			−0.001*	−0.001*		−0.002*				−3.4 × 10^−4^	0.0015
blood in faeces			4.478	4.472		5.285*					4.60
neutrophils (cells µl^−1^)			−0.001	−0.001		−0.001*					−5.7 × 10^−4^
WBC (cells µl^−1^)					−6.30 × 10^−5^			−7.30 × 10^−5^			−6.5 × 10^−5^
conjunctivitis : macrophages (cells µl^−1^)	−0.048										−0.048
mucus: lymphocytes (cells µl^−1^)		0.008									0.008
conjunctivitis: WBC				−2.60 × 10^−4^							−2.60 × 10^−4^
total protein (g l^−1^)								0.156			0.156
blood faeces: WBC									8.98 × 10^−5^		8.98 × 10^−5^

*p = 0.05–0.01, **p < 0.01–0.001.

Only one pup from the group treated early with the antiparasitic drug (ivermectin) developed perineal wounds, while 10 out of the 111 non-treated pups developed perineal wounds in the 2017 breeding season. However, differences were not statistically significant (*χ*^2^ = 1.4, d.f. = 1, *p* = 0.201), probably due to the low statistical power of the experiment (power = 0.22 with a size effect of 0.1, *n* = 146 and d.f. = 1).

## 4. Discussion

The observation of seagull behaviour and close monitoring of marked pups allowed us to confirm that kelp and dolphin gulls are the primary cause of perineal wounds in South American fur seal pups. Although the prevalence of perineal wounds is not very high, and not comparable to the rates of wounding caused by kelp gulls in Southern right whales [11], the actual effect of sea gulls on pups' health could be underestimated, because we only documented cases with a specific physical sequela due to pup–seagull interaction. It is likely that seagull harassment on pups induces stress or other types of more subtle physical effects not detected in our study. There were no significant differences in the prevalence of perineal wounds over seasons, which is in agreement with the lack of significant variation in the number of kelp and dolphin gulls at the fur seal rookery. In Peninsula Valdes, the increased seagull wounding of Southern right whales over the last decades has been associated with a significant increase in the kelp gull populations in the area [11,12].

The presentation of most perineal wounds late in the breeding season (February) coincides with the period of hookworm clearance in fur seal pups [17]. This finding, plus the presence of blood in the faeces and higher hookworm burden in animals with wounds, suggests that seagulls are probably attracted to the higher frequency of defecation in parasitized pups and, probably, the blood in their faeces. Additionally, the hookworm clearance process includes the release of large numbers of nematodes from the pup's intestine (M.S. 2015–2017, personal observations), which could represent an additional food item and stimulus for kelp and dolphin gulls to engage in faeces consumption and picking of the perineal area.

The histological features of the wound biopsies examined support our observations of a primarily traumatic injury, including epidermal laceration, exposure of the dermis and subsequent proliferation of a large amount of granulation tissue. The presence of numerous bacteria in the wounds most probably represents secondary bacterial contamination, given the proximity of the area to the anus and the common presence of faecal material in wounded areas. These bacteria, however, elicit a strong inflammatory response and probably contribute to retarded healing of the wounds. Additionally, the swelling and warmness of the adjacent area, lethargy and marked leukocytosis with left shift in pups with severe wounds suggest that there could have been a systemic compromise of the pup's health status caused by invasion of bacteria into surrounding tissues or to the blood (bacteremia). Nevertheless, these pups also had a significant hookworm infection and the systemic compromise of their health status could have been part of the hookworm haemorrhagic enteritis and bacteremia syndrome, which is the main cause of fur seal pup mortality at Guafo Island [9,18].

The statistical regression performed suggests that anaemia and a higher number of bands are the most consistent factors associated with an increased likelihood of perineal wounds in a pup, although predictors such as ‘blood in faeces’, ‘number of eosinophils' and ‘number of bands’ had a significant effect in some of the models with a delta AIC_c_ of less than 7. The observational and experimental evidence in the studied population indicates that anaemia is caused by hookworm (*Uncinaria* sp*.*) infection [16–18], even though other indicators of hookworm disease, such as the number of parasite eggs in the faeces and bloody diarrhoea, were not significant factors affecting the likelihood of perineal wounds (probably due to low sample size and smaller effect compared with anaemia). In this sense, the presence of severe perineal wounds could be a consequence of anaemia and hookworm disease in the fur seals, because pups that are more affected by this disease (anaemic) probably cannot defend themselves from seagull attacks as well as healthy pups. Our preliminary data on pup behavioural studies indicate that pups with anaemia and more severe hookworm disease tend to socialize and move less than healthy pups, supporting this hypothesis (Montalva and Seguel 2017, unpublished data). On the other hand, the experiment conducted to demonstrate the effect of hookworm parasitism yielded no statistically significant effect of hookworms. However, these results could be biased by the low overall prevalence of perineal ulcers (low size effect), and low sample size, which resulted in low statistical power in our experiment (power = 0.22). Unfortunately, owing to logistical reasons, we were not able to replicate the experiment with a larger sample size. Nevertheless, severe perineal wounds appear to be a significant contributing factor to the deterioration of the health status of these pups; whether they are the cause or consequence of systemic disease processes remains to be determined.

Kelp and dolphin gulls show high trophic plasticity, and their opportunistic nature allows them to switch to dietary sources that offer the best compensation in terms of energy economy [3–5]. On Guafo Island, fur seal carcasses and placentas probably represent the major food sources for seagulls in the rookery. Later in the season, however, when placentas are not available and fur seal pups do not die as often, seagulls may have to look for alternative food sources. In this context, fur seal faeces could be a significant food item for these seagull populations. Among seagulls, coprophagy (the consumption of animal faeces) has been more commonly mentioned in the literature regarding dolphin gulls [19]. It is interesting to note that dolphin gulls are the most common seagull species in the rookery and this species was involved more actively in pup faeces intake and picking of the perineal area. Differences between kelp and dolphin gulls could be related to species behaviour, because dolphin gulls are more aggressive than kelp gulls, or to the fact that other food sources are taken first by kelp gulls because they are almost double the size of dolphin gulls [20]. Therefore, the finding that dolphin gulls wait for kelp gulls to finish their faecal meal before proceeding to feed from pup's perineal area is probably also common when applied to other food sources within the rookery. Indeed, dolphin gulls are more active at trying to find dead pups and sometimes pick the perineal area of sleeping pups in the same manner they do with dead pups, presumably because this guarantees finding food and feeding before the appearance of kelp gulls. These differences in behaviour between dolphin and kelp gulls could also imply a differentiated role in the cause of perineal wounds of fur seal pups. It is possible that dolphin gulls cause more perineal wounds compared with kelp gulls; however, the nature of our observations and assessment of fur seal pups did not allow for testing this hypothesis.

Based on the type and extent of perineal wounds, we consider these lesions collateral damage of seagulls feeding on pups' faeces. Contrary to the case of Cape fur seals in Namibia and Southern right whales in Argentina, kelp and dolphin gulls on Guafo Island do not feed primarily from the tissues of living marine mammals [2,11,12]. In this sense, the ecological interaction between seagull and fur seal pups at Guafo Island could be considered facultative commensalism and parasitism. Some ecologists suggest that true commensalism is non-existent in nature, because all interactions between organisms represent some sort of positive or negative effect for the parts involved, although sometimes effects are very subtle [21]. The case of kelp gull interaction with Southern right whale is an unusual example of parasitism, and was originally recorded as commensalism, in which kelp gulls fed on remains of whale skin floating in the water [22]. Among the factors that could have influenced the change in kelp gull and whale interactions is the mentioned high foraging plasticity of this avian species and the increase in food competition among seagulls because of population growth [11]. Considering the lessons learned from the seagull–whale interaction in Argentina, and our findings in this study, it is important to highlight seagulls as important members of the marine ecosystem and to carefully observe their behaviour and population dynamics because changes in these parameters are likely to affect other species in the marine environment.

Kelp and dolphin gulls cause perineal wounds in South American fur seal pups as a consequence of their feeding on pups’ bloody faeces and expelled nematode parasites. Severe perineal wounds are not very frequent but when present significantly impact the well-being of pups and contribute, with hookworm infection, to the decline in pup health status. In the context of rapid changes in marine ecosystems, additional studies on fur seal and sea gull population dynamics and the drivers of seagull foraging behaviours are warranted to detect and predict the ecosystem-level effects of seagull ecological interactions.

## Supplementary Material

perineal wounds safs.txt

## Data accessibility

The datasets supporting this article have been uploaded as part of the electronic supplementary material.
